# Understanding men’s beliefs and concerns about linking health data in the context of precision medicine

**DOI:** 10.1017/cts.2023.573

**Published:** 2023-06-07

**Authors:** Caitlin G. Allen, Melanie Jefferson, Gayenell Magwood, Cathy Melvin, Oluwole Adeyemi Babatunde, Chanita Hughes Halbert

**Affiliations:** 1 Department of Public Health Sciences, College of Medicine, Medical University of South Carolina, Charleston, SC, USA; 2 Department of Psychiatry and Behavioral Sciences, Hollings Cancer Center, Medical University of South Carolina, Charleston, SC, USA; 3 Department of Biobehavioral Health and Nursing Science, College of Nursing, University of South Carolina, Charleston, SC, USA; 4 Department of Population and Public Health Sciences, University of Southern California, Los Angeles, CA, USA

**Keywords:** Precision medicine, translational science, men’s health, health beliefs, linking data

## Abstract

**Background/Objective:**

Linking data is a critical feature of precision medicine initiatives that involves integrating information from multiple sources to improve researchers’ and clinicians’ ability to deliver care. We have limited understanding of how individuals perceive linking data as it relates to precision medicine. The aim of this study was to identify how sociodemographics, comorbidities, and beliefs about precision medicine influence two outcomes related to linking data: beliefs about linking data and concerns about linking data among men.

**Methods:**

We recruited 124 adult men from primary care practices at a large clinical research university to complete a cross-sectional survey that included questions about sociodemographic characteristics, comorbidities, beliefs, benefits, and limitations of precision medicine, and two outcomes of interest: beliefs about the value of linking data and concerns about linking data. Descriptive statistics, bivariate associations, and multivariable regression were conducted.

**Results:**

Participants had positive beliefs about linking data for precision medicine (M = 4.05/5) and average concern about linking data (M = 2.1/5). Final multivariable models revealed that higher levels of loneliness are associated with more positive beliefs about linking data (β = 0.41, *p* = 0.027). Races other than African American (β = –0.64, *p* = 0.009) and those with lower perceived limitations of precision medicine were less likely to be concerned about linking data (β = –0.75, *p* = 0.0006).

**Conclusion:**

Our results advance the literature about perceptions of linking data for use in clinical and research studies among men. Better understanding of factors associated with more positive perceptions of data linkages could help improve how researchers recruit and engage participants.

## Introduction

Precision medicine is an approach designed to treat and prevent diseases that accounts for variability at the individual level, including genetic, environmental, and lifestyle differences [1,2]. Despite its promise, uptake of precision medicine continues to be low among racial and ethnic minority groups and men, populations that could greatly benefit from advances in precision medicine [3,4]. Since the initial enthusiasm for precision medicine, research has been conducted to identify ways to facilitate the involvement of individuals who do not typically participate in precision medicine interventions (e.g., racial and ethnic minority groups and men). Factors that may influence precision medicine participation include distrust of medical research, understanding motivations of research and medical institutions, lack of awareness and knowledge about studies, concern about exploitation, and racial discrimination [5–7].

While we are beginning to broadly understand and address factors that influence the likelihood of people’s participation in precision medicine initiatives, there has yet to be a critical appraisal of the unique contribution of individuals’ beliefs about linking data on participation in these precision medicine initiatives [8–10]. Data linking is a central feature of precision medicine that allows researchers to have clearer understanding of the cumulative risk of developing disease. As researchers and clinicians generate more information about an individual, these data (often from multiple sources) must be linked together to be most effective. For example, linking information from population registries, the census, and education systems could help improve approaches to delivery of health services [11]. Our prior research indicates that a quarter of individuals would be very likely to donate biospecimens to biobanks [5,6], and others have found positive beliefs and willingness of diverse populations to donate biospecimens for future unplanned use [12]. While these findings indicate high rates of agreement for primary and secondary use of de-identified data among men from racial and ethnic minority groups, there is limited understanding of comfort with usability of data that are linked to identifiable protected health information.

Given the lack of understanding about public perceptions and a dearth of publications about linking data, in the present study, we sought to evaluate aspects of linking data more critically. The aim of this study was to identify how sociodemographics, comorbidities, and beliefs about precision medicine influence two outcomes related to linking data: beliefs and concerns about linking data among men.

## Methods

### Participants

In 2018, the Medical University of South Carolina (MUSC) Transdisciplinary Collaborative Center for Precision Medicine and Minority Men’s Health (MUSC TCC), funded by the National Institute for Minority Health and Health Disparities (NIMHD) and the National Cancer Institute (NCI), began conducting translational research to identify the mechanisms by which psychosocial stressors influence biological processes that are important to the initiation and progression of disease and response to treatment among racially and clinically diverse men. Understanding the nature and distribution of social determinants of health and ethical, legal, and social issues in conducting precision medicine research among minority men are also a focus of the MUSC TCC. Data for the current study comes from male primary care patients between the ages of 21 and 75 years who had medical visits at MUSC practices during the past 5 years. A total of 367 patients were invited through our patient list and self-referred from clinical advertisements. Of those, 13 were ineligible due to being outside of the appropriate time interval for a primary care visit. In total, 354 individuals started the survey and 124 were included in the analysis (35% participation rate).

### Recruitment

We identified patients who met inclusion criteria from the MUSC research data warehouse. All eligible individuals were mailed an invitation letter and were able to opt out of receiving additional research information. Patients that chose to participate were contacted for a one-time cross-sectional survey that included questions about their knowledge, perceptions, and experiences of precision medicine. This survey also included questions about ways that data may be linked for use in precision medicine. Only baseline data from the initial cross-sectional survey are used in the present analysis.

Patients that completed the survey did not receive any formal educational materials about precision medicine or detailed information regarding risks and protections of linking their personal health data for research and clinical care. However, prior to the administration of the survey, patients were provided a brief overview about the importance of precision medicine practices for preventing and treating diseases through the utilization of multiple sources of information to develop more targeted, personalized plans for health care. Further, as part of the survey, participants were provided an additional brief statement about how precision medicine involves linking genetic, environmental, and lifestyle data to develop personalized approaches for both medical care and research prior to their answering questions related to those issues.

### Variables

***Sociodemographic characteristics.*** Sociodemographic characteristics. Self-reported data were collected about race (African American or Black, White or Caucasian, Native American or Aleutian or Eskimo, Asian, Native Hawaiian or Pacific Islander, or Other), ethnicity (Hispanic or Latino), age, marital status (married, divorced, widowed, separated, never married, a member of an unmarried couple, and don't know), employment (not employed, full-time employed, part-time employed, retired, and don't know), level of education (8 or less years of school, some high school, high school graduate/GED, some college, college graduate or beyond, and don't know), income level (less than $20,000, $20,001–$35,000, $35,001–$50,000, $50,001-$75,000, greater than $75,000, and don't know), and health insurance (Yes and No) by self-report using measures from our previous research [13]. These items were collapsed based on the distribution of responses (e.g., African American and Other; married or unmarried; employed, not employed, and retired). We also asked participants about their level of financial strain using a validated item from previous epidemiological research. Specifically, participants were asked: “At the end of the month do you have: some money left over, just enough money left over, or not enough money left over” [14]. We also asked whether participants have previously participated in research studies (yes and no), and about their level of trust in health care providers, with response options of almost all of the time, most of the time, some of the time, and almost none of the time [13].

***Clinical characteristics.*** We asked individuals to share their self-rated overall health status, which included response options: excellent, very good, good, fair, and poor. Next, participants were asked about comorbidities including high blood pressure, heart problems, diabetes, arthritis, high cholesterol, and any other condition (yes and no). These questions were collapsed to include those with at least one condition.

**Social determinants.** We used the Short Form of the Loneliness Scale [15] to measure social isolation. Perceived stress was measured using the four-item version of the Perceived Stress Scale [16]. Ability to adapt (I have been able to adapt when changes occur) was assessed using a five-point Likert scale.

***Beliefs about precision medicine.*** Participants were provided with a definition of precision medicine prior to answering questions about the topic. This definition was developed by the research team and community members as part of MUSC TCC and previously used in research that measured beliefs about emerging technology [17]. This definition read, “Precision medicine is an emerging approach for preventing and treating diseases that uses biological, environmental, and lifestyle information to help develop personalized treatments and procedures. By combining this information, the delivery of medical care will be more personalized as doctors and patients will be able to co-develop targeted plans for prevention, detection and treatment. The goal for precision medicine is to provide the right medical care in the right dose to the right patient at the right time.”

Nine statements focused on beliefs about precision medicine. These statements have previously been used by our study team [5,6]. We report both the average of beliefs across all nine statements and response to each question. All statements included the following level of agreement response options: strongly disagree, disagree, neither disagree/agree, agree, and strongly agree. The statements included, “Precision medicine will improve people’s overall medical care,” “Precision medicine will discriminate against people who are less responsive to medical treatment,” (reverse coded)“Precision medicine will make no difference in people’s lives,” (reverse-coded “Precision medicine should be used as a basis for medical treatment,” “Precision medicine will improve health care,” “Precision medicine is a good way to personalize medical care,” “Precision medicine will limit some people’s access to medical treatment,” (reverse-coded “People like me will not benefit from precision medicine,”(reverse-coded), and “People will not trust precision medicine.” (reverse-coded)

***Benefits of precision medicine.*** Beliefs about the benefits of precision medicine were assessed using five statements, which are reported independently and as an average. These statements have been previously used by our study team [5,6]. Response options included strongly disagree,, disagree, neither agree nor disagree, agree, and strongly agree. The statements were as follows, “My doctor and I would be able to choose medical care that is more likely to be effective,” “I would have more control over the detection, precision, and treatment of diseases,” “I would be able to avoid side effects,” “I would have fewer invasive procedures,” and “I would be able to avoid a trial and error approach to health care.”

***Limitations of precision medicine.*** Limitations of precision medicine included seven statements preciously used by our study team [5,6] The response options included strongly disagree, disagree, neither agree not disagree, agree, and strongly agree. These questions included, “My personal information would be used against me when getting health care,” “The information would be used to deny coverage for a health service that I want or need,” “I would not be able to use personalized strategies because my doctor’s office does not have them,” “I would get information about my health that I did not want to know,” “I would not be able to afford personalized strategies for treating diseases,” “I would not be able to afford personalized strategies for preventing diseases,” and “I would not be able to afford personalized strategies for detecting diseases.”

***Outcomes: beliefs about linking data and concerns about linking data.*** Prior to obtaining participant’s responses about their beliefs about linking data, we also described linking data as: “Precision medicine involves linking genetic, environmental, and lifestyle data to develop personalized approaches for medical care. Please tell me how much you agree with each of the statements about linking data for precision medicine.” Two Likert-style items were used to measure beliefs about linking genetic, environmental, and lifestyle data: (1) beliefs about linking data for precision medicine and (2) concern about linking data. Beliefs about linking data for precision medicine included four statements with response options using a five-point scale ranging from: strongly disagree, disagree, neither agree or disagree, agree, and strongly agree. Both individual and averaged responses are reported. The four statements were, “Information about my genetic, environmental, and lifestyle that is linked together would be used in a harmful way” (reverse-coded, so that lower value means negative or that believe linking data would be used in a harmful way), “Linking information about my genetic, environmental, and lifestyle would help my doctors and me make decisions about detecting disease that were just for me,” “Linking information about my genetic, environmental, and lifestyle would help me and my doctor make decisions about preventing diseases that were just for me,” and “Linking information about my genetic, environmental, and lifestyle would help me and my doctors make decisions about treating diseases that were just for me.”

Concern about linking data was assessed using one question: “How concerned are you about linking data about your genes, environment, and lifestyle?” Responses included: not at all concerned, a little concerned, somewhat concerned, and very concerned.

## Statistical Analyses

All analyses were conducted in SAS version 9.4 software [18]. Descriptive statistics (mean, standard deviation and frequency, percent) were generated to characterize subjects sociodemographics, comorbidities, knowledge of precision medicine, and outcomes of interest (beliefs about linking data and concern about linking data).

We then conducted bivariate analyses to assess associations between the linkage questions and sociodemographic characteristics, precision medicine beliefs, and comorbidities. Both beliefs about linking data and concerns about linking data outcomes were continuous. We conducted bivariate analyses using simple linear regression, with p-values less than *p* = 0.05 considered significant.

Finally, we ran multivariable linear regression models for the two outcomes. Predictor variables included in the final multivariable regression model were selected based on bivariate associations. We used a cutoff of *p* = 0.10 among bivariate associations to identify variable to be included in this final model. The “beliefs about linking data” modelincluded the following predictors: composite beliefs about precision medicine, composite benefits of precision medicine, perceived loneliness, and perceived stress. The “concerns about linking data” model, included race, marital status, income, home ownership status, health insurance status, money at the end of the month, and perceptions about limitations of precision medicine.

## Results

***Description of sociodemographics.*** All individuals who participated in this study were men. Most participants were non-Hispanic White (66.94%) and (Table 1) and 30.65% were African American. The average age was 59.68 (SD = 13.68). Most individuals were married (75.81%) and either employed (38.71%) or retired (45.16%) with a college degree or higher (64.23%) and household income greater than $75,000 (59.83%). Most owned their home (83.61%) and had health insurance (97.56%). Most stated that they had “some money leftover” at the end of the month (61.54%); the majority had participated in a research study (84.48%) and trusted health care providers most of the time (44.54%) or almost all the time (43.7%).

**Table 1.** Sociodemographics and co-morbidities (*N* = 124)

	Frequency or mean	Percent or SD
**Sociodemographic characteristics**		
Race		
African American	38	30.65%
White/Caucasian	83	66.94%
Other	3	2.42%
**Hispanic**		
Yes	3	2.42%
**Age**	**59.68**	**13.68**
**Marital status**		
Married	94	75.81%
Unmarried	30	24.19 %
**Employment status**		
Not employed	20	16.13%
Employed	48	38.71%
Retired	56	45.16%
**Level of education**		
Less than college graduate	44	35.77%
College graduate	79	64.23%
**Income level**		
Less than $75,000	47	40.17%
Greater than $75,000	70	59.83%
**Housing status**		
Own	102	83.61%
Rent	20	16.39%
**Health insurance**		
No	3	2.44%
Yes	120	97.56%
**At the end of the month do you have…**		
Some money leftover	72	61.54%
Just enough money leftover	18	15.38%
Not enough money leftover	27	23.08%
**Previously participated in research study?**		
Yes	106	84.48%
**Trust in health care providers**		
Almost all of the time	52	43.7%
Most of the time	53	44.54%
Some of the time	12	10.08%
Almost none of the time	2	1.68%
**Clinical characteristics**		
**Overall health**		
Excellent	13	10.48%
Very good	36	29.04%
Good	37	29.84%
Fair	29	23.39%
Poor	9	7.26%
**At least one condition**		
Yes	106	85.48%
No	18	14.52%
**Social determinants**		
Loneliness	1.96	0.54
Stress	3.05	0.46
Adapt	4.57	0.8

Overall self-rated health ranged with few ranking themselves as excellent health (10.48%), 29.03% ranked their health as very good, and 29.84% as good health. 85.48% had at least one comorbidity. There were high levels of perceived loneliness (M = 1.96, SD = 0.54, with 1 = hardly ever lonely and 3 = often lonely). Stress levels were average (M = 3.05, SD = 0.46, with 1 indicating lower stress and 5 indicating higher levels of stress). Ability to adapt was high (M = 4.57, SD = 0.8, with 1 indicating less ability to adapt and 5 indicating ability to adapt).

***Description of precision medicine beliefs.*** Overall, individuals had positive beliefs about precision medicine (average across nine items was M = 3.89, SD = 0.5 with 1 = negative beliefs and 5 = positive beliefs). The lowest rated item from this scale was that people will not trust in precision medicine (M = 3.29, SD = 0.89 with 1 = strongly agree that people will not trust precision medicine and 5 = strongly disagree that people will not trust precision medicine). The highest rated item from this scale was the level of agreement that precision medicine will improve health care (M = 4.14, SD = 0.73, 1 = strongly disagree that precision medicine will improve health care, 5 = strongly agree that precision medicine will improve health care).

Individuals also had positive perceptions about the benefits of precision medicine (average across the five items was M = 3.72, SD = 0.47 with 1 = negative benefits and 5 = positive benefits) (Table 2). Participants did not believe they would be able to avoid side effects from precision medicine (M = 3.38, SD = 0.83, 1 = strongly disagree that they would be able to avoid side effects and 5 = strongly agree that they would avoid side effects). Participants felt strongly that precision medicine would allow them to choose the most effective medical care (M = 4.07, SD = 0.51, with 1 = strongly disagree that they could help choose what medical care is most effective and 5 = strongly agree that they could help choose what medical care is most effective). Participants did not see many limitations of precision medicine (M = 3.67, SD = 0.47, 1 = very limited and 5 = not very limited).

**Table 2.** Precision medicine beliefs

	Frequency, Mean	SD, %
**Beliefs about precision medicine**	**3.89**	**0.5**
Precision medicine will improve people’s overall medical care	4.10	0.61
Precision medicine will discriminate against people who are less responsive to medical treatment*	3.55	1
Precision medicine will make no difference in people’s lives*	3.97	0.73
Precision medicine should be used as a basis for medical treatment	3.96	0.74
Precision medicine will improve health care	4.14	0.73
Precision medicine is a good way to personalize medical care	4.17	0.74
Precision medicine will limit some people’s access to medical treatment*	3.61	0.93
People like me will not benefit from precision medicine*	3.91	0.78
People will not trust precision medicine*	3.29	0.89
**Benefits of precision medicine**	**3.72**	**0.47**
My doctor and I would be able to choose medical care that is most likely to be effective	4.07	0.51
I would have more control over the detection, precision, and treatment of diseases	3.89	0.63
I would be able to avoid side effects	3.38	0.83
I would have fewer invasive procedures	3.63	0.69
I would be able to avoid a trial and error approach to health care	3.66	0.80
**Limitations of precision medicine (1=very limited, 5=not very limited)**	**3.67**	**0.64**
My personal information would be used against me when getting health care*	3.85	0.77
The information would be used to deny coverage for a health service that I want or need*	3.56	0.89
I would not be able to use personalized strategies because my doctor’s office does not have them*	3.56	0.8
I would get information about my health that I did not want to know*	3.75	0.9
I would not be able to effort personalized strategies for treating diseases*	3.54	0.86
I would not be able to afford personalized strategies for preventing diseases*	3.59	0.84
I would not be able to afford personalized strategies for detecting diseases*	3.59	0.86

How much have you heard or read about precision medicine? (1–4): 1 = almost nothing, 2 = a little bit, 3 = a fair amount, 4 = a lot Likelihood to participate in precision medicine (1–5): 1 = very unlikely, 2 = unlikely, 3 = neutral, 4 = likely, 5 = very likely.

*Reverse-coded so that lower values (1) are negative and higher values (5) are positive .

***Description of outcomes (beliefs about linking data and concern about linking data).*** Participants reported positive beliefs about linking data (M = 4.04, SD = 1.23, Range of 1 = negative beliefs about linking data and 5 = positive beliefs about linking data). Participants reported average levels of concern about linking data (M = 2.1, SD = 1.11) (Table 3).

**Table 3.** Precision medicine beliefs about linking data and concern about linking data (outcomes)

	Mean	SD
**Beliefs about linking data for precision medicine**	**4.04**	**1.23**
Information about my genetic, environmental, and lifestyle that is linked together would be used in a harmful way*	3.8	0.69
Linking information about my genetic, environmental, and lifestyle would help my doctor and me make decisions about detecting diseases that were just for me	4.14	0.63
Linking information about my genetic, environmental, and lifestyle would help me and my doctor make decisions about preventing diseases that were just for me	4.14	0.6
Linking information about my genetic, environmental, and lifestyle would help me and my doctor make decisions about treating diseases that were just for me	4.11	0.64
**Concern about linking data**		
How concerned are you about linking data about your genes, environment, and lifestyle?	2.1	1.11

*Reverse coded so that lower values (1) are negative and higher values (5) are positive.

***Bivariate associations with beliefs about linking data and concerns about linking data outcomes.*** Those who had positive beliefs about precision medicine (β = 0.3092, *p* = 0.0085) and those who felt precision medicine had benefits were more likely to have positive beliefs about linking data (β = 0.4495, *p* = 0.0018) (Table 4). Those that had higher levels of loneliness were more likely to have positive beliefs about linking data than those with lower levels of loneliness (β = 0.5919, *p* = 0.0052) and individuals with lower perceived stress were more likely to have positive beliefs about linking data than those with lower stress (β = –0.3171, *p* = 0.0388).

**Table 4.** Bivariate analyses for outcomes

	Beliefs about linking data		Concern about linking data	
Sociodemographics	β	*P*-Value	β	*P*-value
*Race*				
African American	ref	ref	ref	ref
Other races	−0.1905	0.4331	−0.522	***0.0148***
*Hispanic*				
Yes	ref	ref	ref	ref
No	−0.6377	0.373	−0.1053	0.8706
Age	−0.0009	0.9083	−0.0093	0.1959
*Marital status*				
Married	ref	ref	ref	ref
Unmarried	0.142	0.5882	−0.4138	0.0763
*Employment status*				
Not employed	ref	ref	ref	ref
Employed	−0.0471	0.8856	−0.1201	0.6873
Retired	−0.1865	0.564	−0.3735	0.2031
*Level of education*				
Less than college graduate	ref	ref	ref	ref
College graduate or beyond	0.1252	0.5962	−0.3393	0.1062
*Income level*				
Less than $75,000 per year	ref	ref	ref	ref
Greater than $75,000 per year	−0.0945	0.6858	−0.388	0.0639
*Housing status*				
Own	ref	ref	ref	ref
Rent	−0.3367	0.2644	0.4687	0.083
*Have health insurance?*				
Yes	ref	ref	ref	ref
No	0.2149	0.7655	−1.2537	***0.0486***
*Amount of money at end of month*				
Some money leftover	ref	ref	ref	ref
Just enough money leftover	0.0697	0.8196	−0.1321	0.6508
Not enough money leftover	0.389	0.1459	0.5055	***0.0446***
Overall health	0.0155	0.8809	0.0287	0.7554
*Previous participation in study*				
Yes	ref	ref	ref	ref
No	0.362	0.1027	0.1137	0.5873
Trust in health care providers	−0.1008	0.4878	−0.2225	0.1203
**Precision medicine beliefs**				
Beliefs about precision medicine	0.3092	***0.0085***	−0.2451	0.3438
Benefits of precision medicine	0.4495	***0.0018***	0.8223	0.3023
Limitations of precision medicine	−0.0984	0.3789	−0.5496	***0.0056***
**Clinical characteristics**				
Overall health	0.0155	0.8809	1.02	0.3989
*Number of comorbidities*				
None	ref	ref	ref	ref
More than one	0.1305	0.6851	−0.2929	0.31
**Social determinants**				
Loneliness	0.5919	***0.0052***	0.1967	0.5519
Stress	−0.3171	***0.0388***	−0.183	0.1883
Adapt	−0.2217	0.1794	−0.1511	0.3214

African American participants were more likely to be concerned about linking data (β = –0.522, *p* = 0.0148). Those without health insurance were less likely than those with health insurance to be concerned about linking data (β = –1.2537, *p* = 0.0486) and compared to those who had “some money leftover” at the end of the month, those with “not enough money leftover” were more likely to be concerned about linking data (β = 0.5055, *p* = 0.0446). Finally, those who perceived precision medicine not to have limitations were less likely to be concerned about linking data (β = –0.5496, *p* = 0.0056).

***Multivariable models.*** Table 5 shows results from multivariable models. Items included in the final model were those that were < 0.10 in bivariate associations. Those that had higher perceived loneliness were more likely to have positive beliefs about linking data (β = 0.4114, *p* = 0.0266).

**Table 5.** Multivariable model

	Beliefs about linking data		Concern about linking data	
	**β**	***P*-value**	**β**	***P*-value**
Belief about precision medicine	−0.2718	0.2044		
Benefit of precision medicine	0.1237	0.6073		
Loneliness	0.4114	***0.0266***		
Stress	−0.049	0.613		
*Race*				
African American			ref	ref
Other races			−0.6644	***0.0088***
*Marital status*				
Married			ref	ref
Unmarried			0.5319	0.1777
*Income*				
Less than $75,000 per year			ref	ref
Greater than $75,000 per year			−0.0327	0.9801
*Housing status*				
Own			ref	ref
Rent			0.3435	0.3505
*Health insurance*				
Yes			ref	ref
No			−0.8675	0.1461
*Money at end of month*				
Some money leftover			ref	ref
Just enough money leftover			−0.0288	0.9297
Not enough money leftover			0.0119	0.9753
Limitations of precision medicine			−0.7501	***0.0006***

When assessing concern about linking data, African American participants were more likely than those from other races to be concerned about linking data (β = –0.6644, *p* = 0.0088), and those with fewer perceived of limitations of precision medicine less likely to be concerned about linking data (β = –0.7501, *p* = 0.0006).

## Discussion

A central feature of precision medicine is the ability to link data about a person’s genetic makeup, lifestyle, and environmental exposures to have a more complete understanding of the cumulative risk of developing diseases that can inform provider decision-making. However, we currently have limited understanding about individual’s beliefs about linking data or concerns about the process of linking data. The current analysis demonstrates men in a primary care sample have positive beliefs about linking data and have a limited amount of concern about linking data on genetic, lifestyle, and environmental characteristics as part of precision medicine strategies.

Loneliness was the only significant predictor of beliefs about linking data in the final model. Increased level of loneliness was associated with more positive perceptions about linking data. Prior literature has suggested that African American’s interest in participating in research is partially driven by motivation to contribute to the broader community and future generations (i.e., altruism). While our outcomes are focused on linking data to support precision medicine in clinical practice, past research has found that those who have higher levels of social isolation may be more interested in and have positive perceptions of linking data for research. These findings have shown that those who participate in research feel more connected to their community or larger research efforts [19,5,20,21]. Additionally, a previous study of retention of African Americans in a randomized controlled trial found that the odds of being retained at all time points in the study was higher among participants who enrolled with a partner into the study (2.95, 95% CI: 1.87–4.65) compared with participants who had no study partner enrolled [22].

Concerns about linking data were associated significantly with racial background. African Americans were more likely to be concerned about linking data than White patients. Distrust in research, health care, and medical or research institutes among racial and ethnic minorities are well documented in the literature [21,23,24]. In genetics research, African Americans have been shown to be concerned about trust, privacy, and the value of genomics [21,24,25]. Our results suggest that there may be similar issues when racial and ethnic minority groups are considering linking different types of personal health information for use in precision medicine initiatives in clinical settings. However, there are currently mixed findings about the impact of these concerns on actual participation. Although distrust may exist, literature has shown a complex tension between these levels of distrust and the need to participate and contribute to research [19,26,27]. Future research could explore the intersection of beliefs about data linkages and actual participation in precision medicine (e.g., an individual participating in population-based genetic screening or treatment that includes precision medicine approaches).

Our findings advance the literature by demonstrating a unique component of data reuse – linking to *identifiable* health information for use in precision medicine. Prior research about data reuse with biorepository data found high rates of agreement for future use of deidentified biospecimens [12]. The use of identified clinical information could present a challenge for participation in precision medicine initiatives and precision-based clinical care. To mitigate this concern for secondary or broader use, researchers have suggested developing honest broker services to share de-identified clinical data to researchers for research only; however, there have not been efforts to address concern about linking data for precision-based clinical care [12].

Addressing concerns about linking data is important in efforts to continue advancing precision medicine initiatives. We found that people who perceived precision medicine to have fewer limitations (e.g., that precision medicine would not result in denial of health coverage, precision medicine would not impact affordability of health care) were less likely to be concerned about data linking. Bolstering understanding of precision medicine and helping further reduce perceived limitations of precision medicine could mitigate concern about data linking. Interventions designed to address concerns may include helping individuals better understand the consent process for participating in precision medicine, directly address limitations of precision medicine, and describe the overall goals of precision medicine approaches. For example, the Partnering Around Cancer Clinical Trials (PACCT) is delivering a multilevel intervention designed to work with patients and participants to increase rates of African American men with prostate cancer to participate in a clinical trial at NCI-designated comprehensive cancer centers. Their approach to improving participation rates is focused on high-quality communication between patients and physicians prior to offering enrollment in a clinical trial [28].

In addition, it could be important to consider where individuals are engaging with health systems. For example, individuals who lack social interaction may be less likely to engage with large medical centers where precision medicine interventions and precision medicine research are often taking place. If individuals are primarily interacting with federally qualified health centers (FQHCs) or community-based clinical settings, they likely do not have exposure to precision medicine or will be as familiar with this approach. One study found significant differences in attendance at clinical appointment by race, with more frequent missed appointments among racial/ethnic minorities compared with non-Hispanic White patients [29]. If racial/ethnic minorities are not engaging in clinical care broadly, this may impact the likelihood they would know about or be engaged in precision medicine interventions. This also reflects a broader issue of how and where precision medicine is likely being implemented (e.g., academic medical centers vs. FQHCs) and accessibility of precision medicine approaches across care settings.

This study is not without limitations. Our sample was drawn from patients who have been to primary care clinics at an academic medical center over the last 5 years and have a relatively high socioeconomic status. Other potential bias includes retention rate, high levels of prior participation in research, and our recruitment strategy focused on primary care settings. Thus, this groups of individuals may have more favorable views of precision medicine than those who do not have direct experience with a large research institution and receive their health care from other sources such as FQHCs or community clinics. Additionally, our sample indicated relatively low overall frequency of hearing about precision medicine, but the majority still had positive perceptions of precision medicine and data linkages. More assessment of participant’s understanding of the meaning of precision medicine and data linkages as well as prior participation in research could help ensure participants are not biased. We recognize the limitations of our cross-sectional survey study design. We focused on individual’s perceptions about data linking at a single time point; however, it is possible that responses could be different in other contexts or if participants were given more detail about the risks of linking data. Finally, only one item was used to assess concern about linking data (“How concerned are you about linking data about your genes, environment, and lifestyle?”). This question only assessed the level of concern (Likert scale 1–4 of not at all concerned to very concerned) and did not capture reasons underlying the concern.

Results from our study can help bolster efforts to improve participation rates among racial and ethnic minorities in precision medicine efforts. Specifically, we consider the levels of understanding and concern about linking data, a critical component of precision medicine. As we continue to advance precision medicine efforts through data linkages, we must be thoughtful in involving racial and ethnic minorities to ensure representation and reduce the likelihood of exacerbating existing health disparities.

## Data Availability

The data that support the findings of this study are available from the corresponding author upon reasonable request.
